# Simple Scores of Fibrosis and Mortality in Patients with NAFLD: A Systematic Review with Meta-Analysis

**DOI:** 10.3390/jcm7080219

**Published:** 2018-08-15

**Authors:** Federico Salomone, Agnieszka Micek, Justyna Godos

**Affiliations:** 1 Division of Gastroenterology, Ospedale di Acireale, Azienda Sanitaria Provinciale di Catania, 95124 Catania, Italy; 2 Department of Nursing Management and Epidemiology Nursing, Jagiellonian University Medical College, 33-332 Krakow, Poland; agnieszka.micek@uj.edu.pl; 3 Department of Biomedical and Biotechnological Sciences (BIOMETEC), University of Catania, 95125 Catania, Italy; justynagodos@gmail.com

**Keywords:** NAFLD fibrosis score, APRI, FIB-4, mortality

## Abstract

Noninvasive simple scores have been validated to assess advanced liver fibrosis in patients with nonalcoholic fatty liver disease (NAFLD). We performed a systematic review with meta-analysis evaluating if NAFLD fibrosis score (NFS), AST to platelet ratio index (APRI), and Fibrosis-4 (FIB-4) score may also predict mortality. PubMed and EMBASE databases were searched until April 2018. Random-effects models were used to calculate pooled RRs of mortality for highest vs. lowest categories of exposure and to perform dose-response meta-analysis. Heterogeneity was assessed using the Q test and *I*^2^ statistic. Overall, eight studies were included in the systematic review; all of the eight studies provided data for NFS, while four provided data for APRI and FIB-4. When comparing the risk estimates for high (>0.676) vs. intermediate + low NFS (≤0.676), we found a nearly fourfold increase in mortality risk, with evidence of heterogeneity (RR = 3.85, 95% CI: 2.08, 7.11; *I*^2^ = 92%). At dose-response meta-analysis, compared to the midpoint of the lowest category of NFS (−2.5), the risk of mortality was about twofold higher for NFS = −0.5 (RR = 2.20, 95% CI: 1.31, 3.70) and more than fivefold higher for NFS = 1.5 (RR = 5.16, 95% CI: 2.02, 13.16). When comparing the risk estimates for high (>1.5) vs. medium + low APRI (≤1.5), we found a higher risk of mortality, without heterogeneity (RR = 3.61, 95% CI: 1.79, 7.28; *I*^2^ = 0%). Comparison of the risk estimates for high (>2.67) vs. medium + low FIB-4 (≤2.67) didn’t reveal a significantly higher risk of mortality, with heterogeneity (RR = 2.27, 95% CI: 0.72, 7.15; *I*^2^ = 85%). Dose-response analysis for APRI and FIB-4 was not considered conclusive due to the low number of studies. Based on the results of our meta-analysis, the measurement of NFS can be considered an accurate tool for the stratification of the risk of death in patients with NAFLD.

## 1. Introduction

Nonalcoholic fatty liver disease (NAFLD) has become a major health problem worldwide because of its potential to evolve into cirrhosis and hepatocellular carcinoma (HCC) [1]. Epidemiological studies from different countries have indicated that fibrosis is the best predictor of liver-related and overall mortality in patients with NAFLD [2]. For this reason, a main goal in the management of NAFLD patients is to identify those at higher risk of clinically significant fibrosis, as also recently underlined in AASLD (American Association for the Study of Liver Diseases) practice guidance and EASL (European Association for the Study of the Liver) guidelines [3,4]. Obviously, liver biopsy is unsuitable for screening of clinically significant fibrosis for a condition that affects at least 30% of adult people in industrialized countries [1]. 

In the last decade, different noninvasive blood-based and imaging-based biomarkers have been developed and validated for the assessment of liver fibrosis in patients with NAFLD [5,6]. Simple blood-based scores including routine parameters can be easily obtained and thus are suitable also in the primary care setting. Among them, the NAFLD fibrosis score (NFS) [7] that can be easily calculated online (http://nafldscore.com/) has shown a good performance for detecting advanced fibrosis and cirrhosis [8]. Excluding patented blood-based scores, other scores that can be easily obtained, and thus are considered simple noninvasive scores, are APRI, FIB-4, BARD, and Forns [5]. 

Besides prediction of clinically significant fibrosis, in the last five years, retrospective and prospective studies have evaluated the predictive value of simple scores of fibrosis for clinically relevant outcomes such as liver events/transplantation and general mortality [9,10,11,12,13,14,15,16]. Here we performed a systematic review with meta-analysis of these studies.

## 2. Methods

### 2.1. Search Strategy

We followed the PRISMA (Preferred Reporting Items for Systematic Reviews and Meta-Analyses) checklist [17] for transparent reporting in systematic reviews and meta-analyses (Supplementary Figure S1). A comprehensive literature search was conducted using two databases: PubMed and EMBASE; databases were screened up to April 2018, with a restriction to publications in English. The following search terms were used: (NAFLD OR nonalcoholic fatty liver disease OR non-alcoholic fatty liver disease OR fatty liver OR liver steatosis OR NASH OR nonalcoholic steatohepatitis OR non-alcoholic steatohepatitis OR steatohepatitis) AND (mortality OR survival OR death) AND (cohort OR cross-sectional OR longitudinal OR retrospective). Two authors (FS, JG) independently screened titles and abstracts of all identified studies. Discrepancies were resolved by consensus. Additionally, the reference lists of retrieved articles were hand searched to identify studies not previously detected. When duplicate reports on the same cohort were identified, the one with the largest number of cases/entire cohort or with the longest follow-up for endpoint of interest was included. Eligibility criteria for study inclusion in the meta-analysis were based on the following criteria: (i) study design: cohort study with prospective or retrospective design; (ii) population with a diagnosis of NAFLD; (iii) mortality as the endpoint outcome; (iv) the measure of association (relative risk, odds ratio, hazard ratio) with 95% confidence interval between scores and mortality.

### 2.2. Data Extraction

Using a standardized extraction form, data were abstracted from all identified studies. The following information was obtained from each article: (1) first author name; (2) year of publication; (3) study design; (4) country; (5) name or type of cohort; (6) age range of the study population at baseline; (7) tool used for NAFLD diagnosis; (8) follow-up period; (9) number of cases; (10) dose for each category of exposure; (11) relative risks or hazard ratios, with 95% CI; (12) covariates used in adjustments. 

### 2.3. Statistical Analysis

In this meta-analysis, ORs and HRs were deemed equivalent to RRs. Random-effects models were used to calculate pooled RRs (with 95% CIs) of mortality for highest vs. lowest categories of exposure and for dose-response analysis [18] (Supplementary methods). Heterogeneity was assessed using the Q test and *I*^2^ statistic. The significance for the Q test was defined as *p* < 0.10. The *I*^2^ statistic represents the sum of total variation that could be attributed to heterogeneity. *I*^2^ values ≤ 25%, 25–50%, 50–75% and >75% indicated no, small, moderate, and high heterogeneity, respectively. A sensitivity analysis, where possible, was conducted by excluding one study at the time to assess the stability of results. Subgroup analyses were conducted by study design, geographical location, tool used for NAFLD diagnosis, and adjustment for confounding factors. Publication bias was assessed by visual observation of funnel plot (Supplementary Figure S2). All analyses were performed with Review Manager (RevMan) version 5.2.

## 3. Results

**Figure 1:**
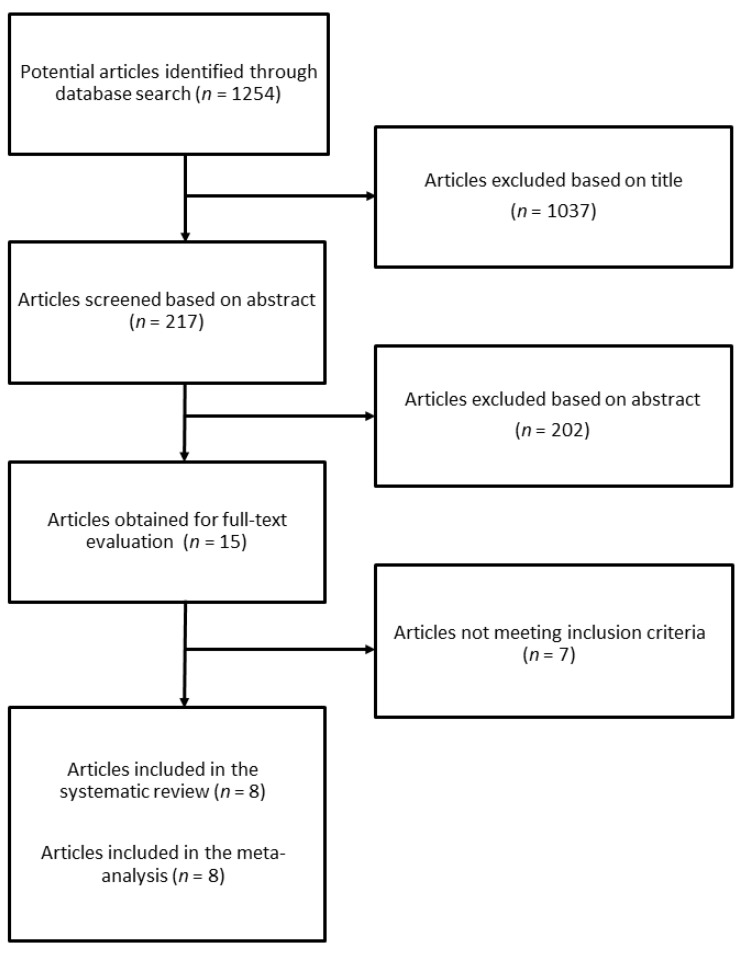
Process selection of relevant studies reporting on the association between simple scores of fibrosis and mortality in NAFLD patients.

The search identified 1254 studies, of which 1037 were excluded after reviewing the title, and 202 after reviewing the abstract (Figure 1). Of the 15 publications selected for the evaluation of full-text article, 7 were excluded for the following reasons: (1) article did not provide risk with confidence intervals; (2) article did not have prospective or retrospective design; (3) article provided data for the association between NAFLD and mortality, but not for scores; (4) mortality was not the endpoint outcome; (5) article reported on other scores/indexes.

For the systematic review on the association between noninvasive scores of fibrosis and mortality in NAFLD patients, eight studies were eligible [9,10,11,12,13,14,15,16]. All the eight articles provided data for NFS [9,10,11,12,13,14,15,16], and 4 articles for APRI and FIB-4 [9,11,13,14]. Selected characteristics of the studies included in the systematic review are described in Table 1 and Supplementary Table S1. Among the eight studies, only one study provided data for mortality for BARD [11] and one for Forns [14], and thus these two scores were not considered for the meta-analysis. 

Briefly, 4 studies were prospective cohorts [9,14,15,16], while others were retrospective studies [10,11,12,13]. Three studies included patients with biopsy-proven NAFLD [10,11,13]; two studies included patients with liver steatosis diagnosed by ultrasonography [9,12] and two studies included patients with a diagnosis of NAFLD based on serum markers [14,15]; one study did not assess the presence of NAFLD [16]. Six studies adjusted for potential confounding factors [9,11,13,14,15,16] and two did not [10,12]. Five studies were conducted in North America [9,10,13,14,15], two in Asia [12,16], and one study was multicountry [11]. The median follow-up ranged from 3 to about 19 years.

### 3.1. NAFLD Fibrosis Score

**Figure 2:**
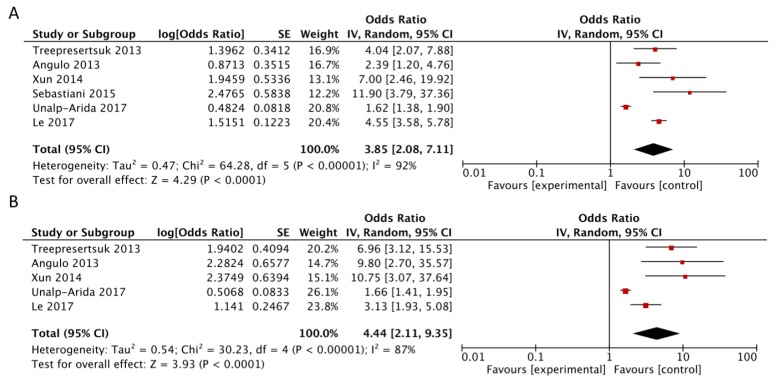
Forest plot of summary relative risks (RRs) of mortality (**A**) for high versus low–intermediate NAFLD fibrosis score, and (**B**) for high versus low NAFLD fibrosis score.

Among the eight studies, six provided data for the high vs. intermediate-low analysis [10,11,12,13,14,15]. The study by Kim et al. [9] was excluded because the same NHANES cohort with a longer follow-up was analyzed by Unalp-Arida five years later [14]. The study by Yoshihisa et al. [16] was included in the dose-response analysis but was excluded from the high vs. intermediate + low analysis because it used cut-points for NFS that were different from cut-points used in all other studies. When comparing the risk estimates for high (>0.676) vs. intermediate-low NFS (≤0.676), we found a nearly fourfold increase in risk of mortality (RR = 3.85, 95% CI: 2.08, 7.11; *I*^2^ = 92%), with evidence of heterogeneity (Figure 2, Panel A). The results were similar in the analysis comparing high (>0.676) vs. low NFS (<−1.455) (RR = 4.44, 95% CI: 2.11, 9.35; *I*^2^ = 87%) (Figure 2, Panel B). In this latter analysis, Sebastiani et al. [13] was not included because it reported only NFS > 0.676 vs. ≤ 0.676.

Heterogeneity was due to the results of Unalp-Arida’s study [14] that reported substantial lower risk estimates compared to other studies; a potential reason for such findings is that authors assessed the predictive value of noninvasive scores in a general NHANES population of virus-negative individuals [14]. However, after exclusion of this study in the sensitivity analysis, overall risk estimate remained significant and heterogeneity decreased (RR = 4.49, 95% CI: 3.08, 6.54; *I*^2^ = 41%, P_heterogeneity_ = 0.15). The subgroup analysis showed a stronger positive association for the studies that included cases diagnosed using liver biopsy (RR = 4.33, 95% CI: 1.99, 9.43), studies with retrospective design (RR = 4.72, 95% CI: 2.50, 8.93), and studies that adjusted for confounding factors (RR = 4.45, 95% CI: 2.35, 8.43), with evidence of lower heterogeneity (Table 2).

**Figure 3:**
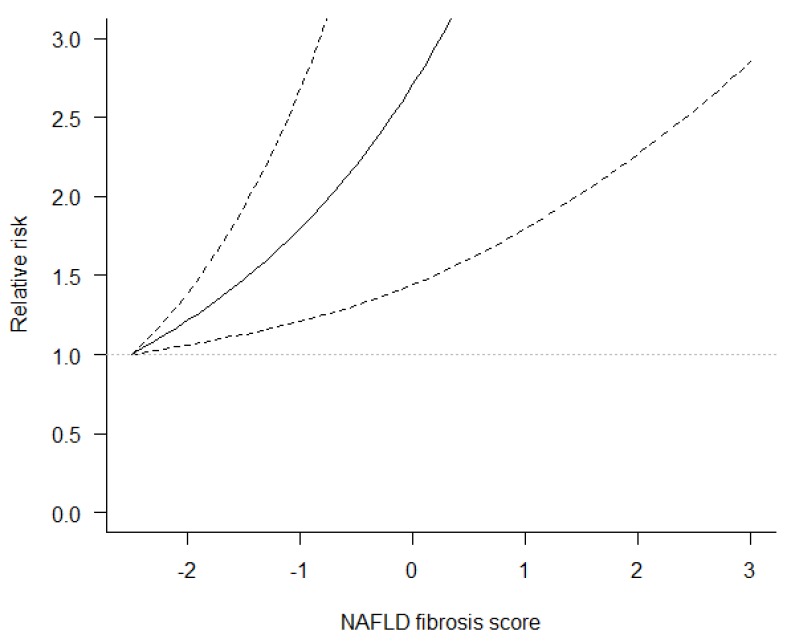
Dose-response association between NAFLD fibrosis score and mortality risk in NAFLD patients. Solid lines represent risk ratio, dashed lines represent 95% confidence intervals.

Data from six studies [10,11,12,14,15,16] were used to perform dose-response meta-analysis of risk estimates for NFS. The analysis showed that compared to the midpoint of the lowest category of NFS (−2.5), the risk of mortality was about twofold higher for NFS = −0.5 (RR = 2.20, 95% CI: 1.31, 3.70) and more than fivefold higher for NFS = 1.5 (RR = 5.16, 95% CI: 2.02, 13.16) (Figure 3) (Supplementary Table S2).

### 3.2. APRI and FIB4

**Figure 4:**
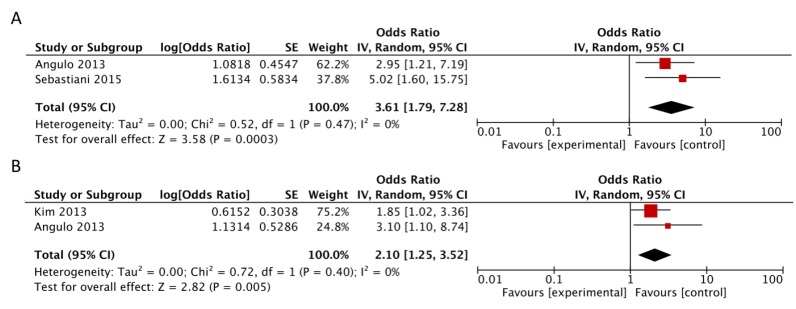
Forest plot of summary relative risks (RRs) of mortality (**A**) for high versus low-intermediate APRI and (**B**) for high versus low APRI.

The meta-analysis of risk estimates for the other two scores was limited by the small number of studies includable. Concerning APRI, studies by Kim et al. [9] and Angulo et al. [11] provided data in three categories of exposure: high (>1.5), medium (0.5–1.5) and low (<0.5), as in the study that originally proposed the score [19]. Sebastiani et al. [13] considered only two categories of exposure: high (>1.5) and medium-low (≤1.5). Differently, Unalp-Arida et al. [14] provided two other categories: medium-high (>0.5) vs. low (<0.5). Thus, considering the high vs. intermediate-low analysis for APRI, we were able to include only data from Angulo et al. [11] and Sebastiani et al. [13] but not from Kim et al. [9] because this latter study didn’t provide the numbers of cases, controls, covariate-adjusted OR, and CIs that are required to pool the categories medium (0.5–1.5) and low (<0.5) together to obtain the category <1.5 by the Hamling method. With data from the two studies [11,13], when comparing high (>1.5) vs. intermediate + low APRI (≤1.5), we found a higher mortality risk with no evidence of heterogeneity (RR = 3.61, 95% CI: 1.79, 7.28; *I*^2^ = 0%) (Figure 4, Panel A).

In the analysis of high vs. low APRI, we were able to include only Angulo et al. [11] and Kim et al. [9] because, as previously stated, Sebastiani et al. [13] considered only two categories of exposure: high (>1.5) and medium-low (≤1.5) and again it was not possible to include data from Unalp-Arida et al. because they used different cut-points from [19] and from the other studies used for this meta-analysis. With data from the two studies [9,11], when comparing the risk estimates for high (>1.5) vs. low APRI (<0.5), we found a twofold increased risk of mortality, without heterogeneity (RR = 2.10, 95% CI: 1.25, 3.52; *I*^2^ = 0%) (Figure 4, Panel B).

**Figure 5:**
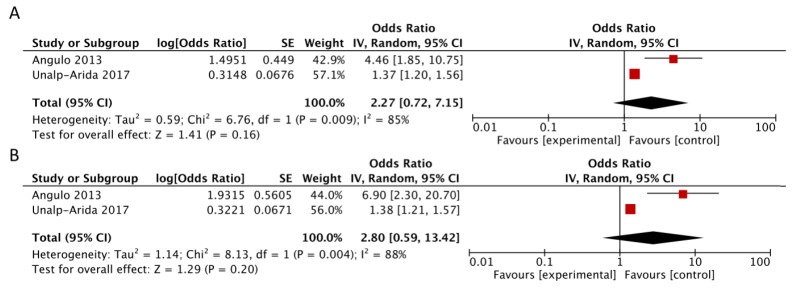
Forest plot of summary relative risks (RRs) of mortality (**A**) for high versus low-intermediate FIB-4 and (**B**) for high versus low FIB-4.

Concerning FIB-4, studies by Kim et al. [9], Angulo et al. [11], and Unalp-Arida et al. [14] provided data in three categories of exposure: high (>2.67), medium (1.30–2.67), and low (<1.30), as in the study that validated this score in the NAFLD setting [20]. Sebastiani et al. [13] considered two categories of exposure (>3.25 vs. ≤3.25) in contrast with the other three available studies and thus was not included. Since Unalp-Arida et al. reported data from Kim et al. with a longer follow-up, only data from [11] and [14] were available for comparison of high (>2.67) vs. medium-low FIB-4 (≤2.67) that didn’t reveal a significantly higher risk of mortality, with evidence of heterogeneity (RR = 2.27, 95% CI: 0.72, 7.15; *I*^2^ = 85%, *p* = 0.009) (Figure 5, Panel A). Similarly, comparison of the risk estimates for high (>2.67) vs. low FIB-4 (<1.30) didn’t show a higher risk, with evidence of heterogeneity (RR = 2.80, 95% CI: 0.59, 13.42; *I*^2^ = 88%) (Figure 5, Panel B). 

Dose-response meta-analysis of risk estimates was possible only with data from Angulo et al. [11] for APRI and from Angulo et al. and Unalp-Arida et al. [11,14] for FIB-4 score because of missing data on cases and non-cases in the remaining studies. Therefore, dose-response analysis for these two scores have low power and findings cannot be considered conclusive (Supplementary Table S2; Supplementary Figure S2).

## 4. Discussion

In this study, we meta-analyzed retrospective and prospective studies conducted so far, reporting data of association between values of NFS, APRI, and FIB-4 with mortality of patients with NAFLD. Our meta-analysis shows that an NFS > 0.676 is associated with about a fourfold higher risk of death as compared with a low or nonhigh value and NFS is associated with higher risk of mortality in a dose-response manner.

Among studies included in this systematic review, the study by Kim et al. [9] was the first that assessed the ability of simple noninvasive scores of fibrosis in predicting mortality. By analyzing data from the National Health and Nutrition Examination Survey (NHANES), the authors showed that after a median follow of 14.5, among a population of 11,154 individuals with an ultrasonographic diagnosis of liver steatosis, only those with high NFS, APRI, and FIB-4 displayed an increase of mortality with adjustment for several established predictors of mortality. Successively, Treeprasertsuk et al. [10] reviewed retrospectively data from a histologically characterized cohort from the Mayo Clinic and showed that a high NFS predicted all-cause mortality and cardiac and liver complications after a mean follow-up of 12 years, although significance of their findings was limited by the absence of adjustments. The first study considering an international, multicenter cohort was from Angulo et al. [11], who found that high APRI and FIB-4 and both intermediate and high NFS were able to predict death and liver-related events in a biopsy-proven cohort of 320 patients with a follow-up of almost 9 years even after adjustments for several parameters. Xun et al. [12] confirmed the predictive value of NFS, FIB-4, and APRI for all-cause mortality in Chinese patients after a 6.6-year median follow-up although without any adjustment.

Sebastiani et al. [13] evaluated retrospectively a well-characterized Canadian cohort of 148 patients with available liver biopsies and hepatic venous pressure gradient (HVPG) measures and found that APRI > 1.5, FIB-4 > 3.25, and NFS > 0.676 did not significantly differ from histological grading and HVPG in predicting clinical outcomes including death and liver-related complications. Recently, three studies reported simple noninvasive scores’ predictive value for mortality with a prospective design. In the bigger population-based study conducted so far, Unalp-Arida and Ruhl [14] analyzed data from almost 15,000 viral hepatitis-negative adult participants in the third NHANES, 1988–1994, and found an increased overall and liver-related mortality in individuals with a high APRI, FIB-4, Forns, or NFS over a 19.3-year median follow-up, after adjustment for age. Similarly, Le et al. [15] analyzed prospectively data from NHANES 1999–2012 and reported data from 1936 individuals with NAFLD as diagnosed by the US Fatty Liver Index. Authors found that only patients with NFS > 0.676 had a higher risker of all-cause mortality. Interestingly, this is in line with Yoshihisa et al. [16], who investigated the association of NFS with mortality in a prospective cohort of 492 patients followed-up for heart failure with preserved ejection fraction and found a higher risk of death with higher quartiles of NFS. It is well established that cardiovascular diseases are the main cause of death in patients with NAFLD [2] because of the proinflammatory and atherogenic *milieu* associated with liver steatosis and fibrosis.

Besides simple scores, in the last years, several other noninvasive approaches to assess fibrosis in the daily clinical practice have been validated in NAFLD [21], including patented blood tests such as Enhanced Liver Fibrosis (ELF) [22] or imaging-based techniques such as elastography [6]. A recent meta-analysis has shown that magnetic resonance and shear wave elastography display the highest diagnostic accuracy for staging fibrosis in NAFLD patients [8]. However, imaging-based techniques are not widely available in clinical practice among general practitioners or primary healthcare providers. Vibration-controlled transient elastography as assessed by Fibroscan™ has become a main diagnostic tool to assess fibrosis in NAFLD, although its diagnostic accuracy is limited by steatosis degree and other factors [8]. However, Fibroscan™ is almost exclusively diffused in the hospital setting, at least in Italy, and thus the access to elastography needs to be filtered by primary care physicians. Consistently, although several studies from the US are showing the elevated diagnostic accuracy of MRI elastography for the assessment of fibrosis [6], it is reasonable that this sophisticated tool may be reserved for clinical trials at least for the next years. By contrast, simple scores of fibrosis are widely used because they are based on common laboratory parameters alone, such as it is for APRI, or combined with age and body mass index, such as it is for the NFS. Results of our meta-analysis show that there is solid evidence to recommend the clinical use of NFS because of its dose-response association with mortality, whereas there is less evidence for APRI and not enough evidence so far to recommend FIB-4. For this reason, we believe that NFS calculation should be constantly performed to screen which patients should undergo elastography or imaging-based techniques to assess fibrosis. This may be particularly important in non-hepatological contexts (such as general practitioners, diabetologists, and cardiologists) in which elastography or more sophisticated techniques are not widely available.

## 5. Conclusions

In conclusion, results from our meta-analysis suggest that the measurement of NFS can be considered an accurate tool for the stratification of the risk of death and thus should be routinely assessed as a screening test for fibrosis in NAFLD patients.

## Figures and Tables

**Table 1:** Main features of the 8 studies included in the systematic review (NA: not avalaible).

Author, Year, Country	Cohort, Study Design	Number of Participants	Age	Male Gender	Follow-up Duration	NAFLD Diagnosis
Kim, 2013, USA	Population-based, prospective	4081 NAFLD individuals with available APRI, FIB-4, NFS	45.5 years (mean)	50.3%	14.5 years (median)	Ultrasonography
Treeprasertsuk, 2013, USA	Community-based, retrospective	302 NAFLD individuals with available NFS	47.3 years (mean)	44%	12 years (mean)	Liver biopsy
Angulo, 2013, Multi-country	Hospital-based, multicenter, retrospective	320 NAFLD individuals with available APRI, FIB-4, NFS	52 years (median)	43%	8.7 years (104.8 months) (median)	Liver biopsy
Xun, 2014, China	Hospital-based single center, retrospective	180 NAFLD individuals with available APRI, FIB-4, NFS	39 years (median)	53.3%	6.6 years (median)	Ultrasonography
Sebastiani, 2015, Canada	Hospital-based single center, retrospective cohort	148 NAFLD individuals with available NFS	49.5 years (mean)	69.6%	5 years (median)	Liver biopsy
Unalp-Arida, 2017, USA	Population-based, prospective	14841 individuals for APRI and FIB-4, 14741 for NFS	NA	NA	19.3 years (median)	Negative for viral hepatitis markers
Le, 2017, USA	Population-based, prospective	1936 NAFLD individuals with available NFS	53.2 years (mean)	59.2%	13 years (up to)	United States Fatty Liver Index (USFLI)
Yoshihisa, 2017, Japan	Hospital-based single center, prospective	492 patients with heart failure and preserved ejection fraction	NA	NA	3.0 years (1096 days) (mean)	Not assessed

**Table 2:** Subgroup analysis of the six studies included in the meta-analysis of NFS and mortality.

Subgroup	No. of Studies	RR (95% CI)	*I* ^2^	*P_heterogeneity_*
Total	6	3.85 (2.08, 7.11)	92%	<0.00001
Study design				
Prospective	2	2.70 (0.98, 7.44)	98%	<0.00001
Retrospective	4	4.72 (2.50, 8.93)	55%	0.08
Geographical location				
North America	4	3.88 (1.80, 8.37)	95%	<0.00001
Asia	1	7.00 (2.46, 19.92)	NA	NA
Multicountry	1	2.39 (1.20–4.76)	NA	NA
Liver biopsy				
Yes	3	4.33 (1.99, 9.43)	64%	0.06
No	3	3.43 (1.42, 8.28)	96%	<0.00001
Adjustment for confounding factors				
Yes	3	4.45 (2.35, 8.43)	67%	0.05
No	3	3.23 (1.32, 7.91)	85%	0.001

## Supplementary Materials

The following are available online at http://www.mdpi.com/2077-0383/7/8/219/s1, Table S1: Total number of participants and number of cases (deaths) for each study included in the meta-analysis evaluating the risk of mortality according to NAFLD fibrosis score, APRI and FIB-4 values, Table S2: Dose-response meta-analysis using splines with knots at quartiles (0.25, 0.50, 0.75 quartiles) assessing the risk of mortality according to NAFLD fibrosis score, APRI and FIB-4 values, Figure S1: PRISMA (Preferred Reporting Items for Systematic Reviews and Meta-Analyses) checklist, Figure S2: Funnel plots for mortality risk in NAFLD patients: (A) for the high versus low (reference) category of NAFLD fibrosis score, (B) for the high versus intermediate/low category of NAFLD fibrosis score, (C) for the high versus low (reference) category of APRI, (D) for the high versus intermediate/low category of APRI, (E) for the high versus low (reference) category of FIB4, (F) for the high versus intermediate/low category of FIB4, Figure S3: Dose-response association between APRI, FIB-4 and mortality risk in NAFLD patients. Solid lines represent risk ratio, dashed lines represent 95% confidence intervals.
