# Supplementary material for: Simple Scores of Fibrosis and Mortality in Patients with NAFLD: A Systematic Review with Meta-Analysis
Source: J Clin Med. 2018 Aug 15;7(8):219. doi: 10.3390/jcm7080219 (PMC6111765; doi:10.3390/jcm7080219)

Methods

Statistical analysis

Dose-response meta-analysis was modeled by using restricted cubic splines with 3 knots at fixed percentiles (25%, 50%, and 75%) of the distribution.

In order to combine categories of exposure to obtain identical categories across considered studies for each type of score, approach set out in Hamling et al was used [Hamling J, Lee P, Weitkunat R, Ambuhl M. Facilitating meta-analyses by deriving relative effect and precision estimates for alternative comparisons from a set of estimates presented by exposure level + or disease category. Statistics in medicine 2008;27(7):954-70. doi: 10.1002/sim.3013.].

Briefly, the standard errors and confidence intervals were re-calculated consistently for each rescaled risk estimate based on the adjusted results presented in the papers. To perform dose-response meta-analysis data on the level of NFS, APRI and FIB-4 score, distributions of cases and participants (when available), and ORs/HRs with 95% CIs for ≥3 categories of score were extracted. The midpoint of the range in each category of each score was assigned to the corresponding OR/HR with the 95% CI for each study. When the highest category was open ended, we assumed the width of the category to be the same as the adjacent category. When the lowest category was open ended, we set the lower boundary to zero.

Supplementary table 1. Total number of participants and number of cases (deaths) for each study included in the meta-analysis evaluating the risk of mortality according to NAFLD fibrosis score, APRI and FIB-4 values

NAFLD fibrosis score

Author, year	Score categories	n	Cases	Non cases
Treeprasertsuk, 2013	< -1.455	181	12	169
"	-1.455 to 0.676	108	21	87
"	> 0.676	13	6	7
Angulo, 2013	< -1.455	125	5	120
"	-1.455 to 0.676	120	20	100
"	> 0.676	75	16	59
Xun, 2014	< -1.455	129	4	125
"	-1.455 to 0.676	39	4	35
"	> 0.676	12	4	8
Sebastiani, 2015	≤ 0.676	NA	NA	NA
"	> 0.676	NA	NA	NA
Le, 2017	< -1.455	739	21	718
"	-1.455 to 0.676	919	107	812
"	> 0.676	245	86	159
Unalp-Arida, 2017	< -1.455	9073	1318	7755
"	-1.455 to 0.676	4413	2328	2085
"	> 0.676	1255	1032	223

NA, not avalaible

APRI

Author, year	Score categories	n	Cases	Non cases
Kim, 2013	< 0.5	NA	NA	NA
"	0.5 to 1.5	NA	NA	NA
"	> 1.5	NA	NA	NA
Angulo, 2013	< 0.5	93	9	84
"	0.5 to 1.5	169	17	152
"	> 1.5	58	15	43
Sebastiani, 2015	< 1.5	NA	NA	NA
"	> 1.5	NA	NA	NA

NA, not avalaible

FIB-4

Author, year	Score categories	n	Cases	Non cases
Kim, 2013	< 1.3	NA	NA	NA
“	1.3 to 2.67	NA	NA	NA
“	> 2.67	NA	NA	NA
Angulo, 2013	< 1.3	111	6	105
“	1.3 to 2.67	117	14	103
“	> 2.67	92	21	71
Unalp-Arida, 2017	< 1.3	10824	1881	8943
“	1.3 to 2.67	3416	2317	1099
“	> 2.67	601	524	77

NA, not available

Supplementary table 2. Dose-response meta-analysis using splines with knots at quartiles (0.25, 0.50, 0.75 quartiles) assessing the risk of mortality according to NAFLD fibrosis score, APRI and FIB-4 values.

Score	Dose category	Dose midpoint	RR (95% CI)	Number of studies	I^2	p heterogeneity
NAFLD fibrosis score						
	< -1.455	-2.5	1.00 (ref.)	6	93.69	< 0.01
	-1.455 to 0.676	-0.5	2.20 (1.31-3.70)			
	> 0.676	1.5	5.16 (2.02-13.16)			
APRI						
	< 0.5	0	1.00 (ref.)	1	-	-
	0.5 to 1.5	1	1.11 (0.3-4.11)			
	> 1.5	2	3.14 (0.88-11.14)			
FIB-4						
	< 1.3	0.5	1.00 (ref.)	2	78.8	0.01
	1.3 to 2.67	2	1.39 (0.67-2.89)			
	> 2.67	3.5	3.04 (0.51-18.12)			

Supplementary figure 1. PRISMA (Preferred Reporting Items for Systematic Reviews and Meta-Analyses) checklist

Section/topic	#	Checklist item	Reported on page #
**TITLE**			
Title	1	Identify the report as a systematic review, meta-analysis, or both.	1
**ABSTRACT**			
Structured summary	2	Provide a structured summary including, as applicable: background; objectives; data sources; study eligibility criteria, participants, and interventions; study appraisal and synthesis methods; results; limitations; conclusions and implications of key findings; systematic review registration number.	1
**INTRODUCTION**			
Rationale	3	Describe the rationale for the review in the context of what is already known.	1,2
Objectives	4	Provide an explicit statement of questions being addressed with reference to participants, interventions, comparisons, outcomes, and study design (PICOS).	2
**METHODS**			
Protocol and registration	5	Indicate if a review protocol exists, if and where it can be accessed (e.g., Web address), and, if available, provide registration information including registration number.	2
Eligibility criteria	6	Specify study characteristics (e.g., PICOS, length of follow-up) and report characteristics (e.g., years considered, language, publication status) used as criteria for eligibility, giving rationale.	2
Information sources	7	Describe all information sources (e.g., databases with dates of coverage, contact with study authors to identify additional studies) in the search and date last searched.	2
Search	8	Present full electronic search strategy for at least one database, including any limits used, such that it could be repeated.	2
Study selection	9	State the process for selecting studies (i.e., screening, eligibility, included in systematic review, and, if applicable, included in the meta-analysis).	2
Data collection process	10	Describe method of data extraction from reports (e.g., piloted forms, independently, in duplicate) and any processes for obtaining and confirming data from investigators.	2
Data items	11	List and define all variables for which data were sought (e.g., PICOS, funding sources) and any assumptions and simplifications made.	2
Risk of bias in individual studies	12	Describe methods used for assessing risk of bias of individual studies (including specification of whether this was done at the study or outcome level), and how this information is to be used in any data synthesis.	NA
Summary measures	13	State the principal summary measures (e.g., risk ratio, difference in means).	2,3
Synthesis of results	14	Describe the methods of handling data and combining results of studies, if done, including measures of consistency (e.g., I^2) for each meta-analysis.	2,3
Risk of bias across studies	15	Specify any assessment of risk of bias that may affect the cumulative evidence (e.g., publication bias, selective reporting within studies).	NA
Additional analyses	16	Describe methods of additional analyses (e.g., sensitivity or subgroup analyses, meta-regression), if done, indicating which were pre-specified.	2,3
**RESULTS**			
Study selection	17	Give numbers of studies screened, assessed for eligibility, and included in the review, with reasons for exclusions at each stage, ideally with a flow diagram.	3, Figure 1
Study characteristics	18	For each study, present characteristics for which data were extracted (e.g., study size, PICOS, follow-up period) and provide the citations.	1,2, Table 1
Risk of bias within	19	Present data on risk of bias of each study and, if available, any outcome level	NA

studies		assessment (see item 12).	
Results of individual studies	20	For all outcomes considered (benefits or harms), present, for each study: (a) simple summary data for each intervention group (b) effect estimates and confidence intervals, ideally with a forest plot.	4-7, Figure 2, Figure 4, Figure 5
Synthesis of results	21	Present results of each meta-analysis done, including confidence intervals and measures of consistency.	4-7, Figure 2, Figure 4, Figure 5
Risk of bias across studies	22	Present results of any assessment of risk of bias across studies (see Item 15).	NA
Additional analysis	23	Give results of additional analyses, if done (e.g., sensitivity or subgroup analyses, meta-regression [see Item 16]).	Table 2, Figure 3
**DISCUSSION**			
Summary of evidence	24	Summarize the main findings including the strength of evidence for each main outcome; consider their relevance to key groups (e.g., healthcare providers, users, and policy makers).	7,8
Limitations	25	Discuss limitations at study and outcome level (e.g., risk of bias), and at review-level (e.g., incomplete retrieval of identified research, reporting bias).	NA
Conclusions	26	Provide a general interpretation of the results in the context of other evidence, and implications for future research.	9
**FUNDING**			
Funding	27	Describe sources of funding for the systematic review and other support (e.g., supply of data); role of funders for the systematic review.	9

Supplementary figure 2. Funnel plots for mortality risk in NAFLD patients: A) for the high versus low (reference) category of NAFLD fibrosis score, B) for the high versus intermediate/low category of NAFLD fibrosis score, C) for the high versus low (reference) category of APRI, D) for the high versus intermediate/low category of APRI, E) for the high versus low (reference) category of FIB4, F) for the high versus intermediate/low category of FIB4.

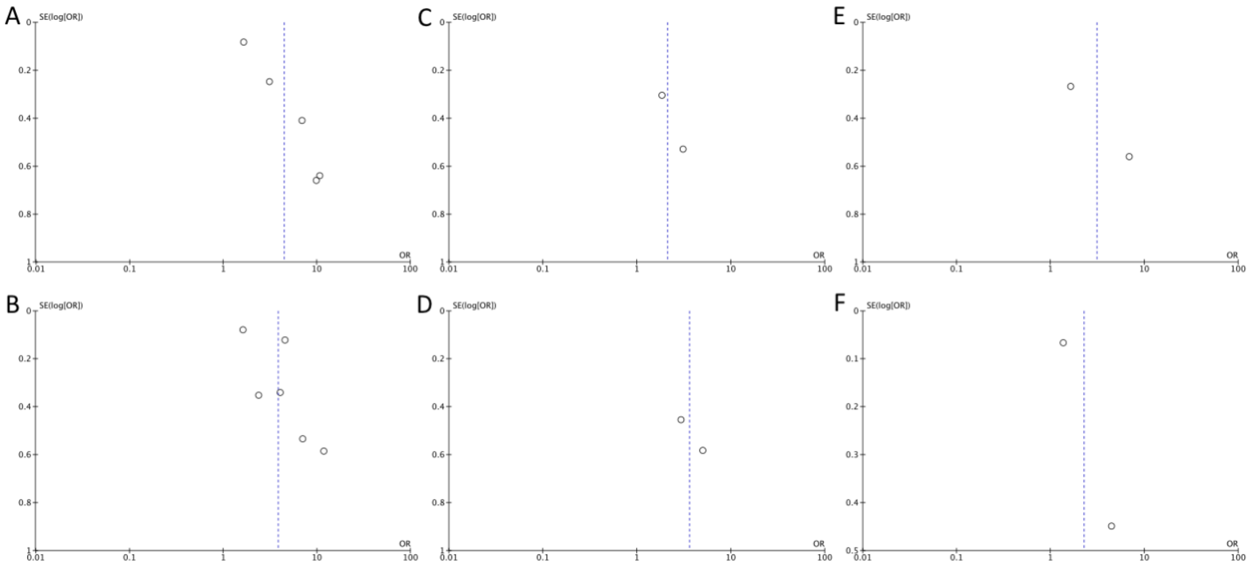

Supplementary figure 3. Dose-response association between APRI, FIB-4 and mortality risk in NAFLD patients. Solid lines represent risk ratio, dashed lines represent 95% confidence intervals.

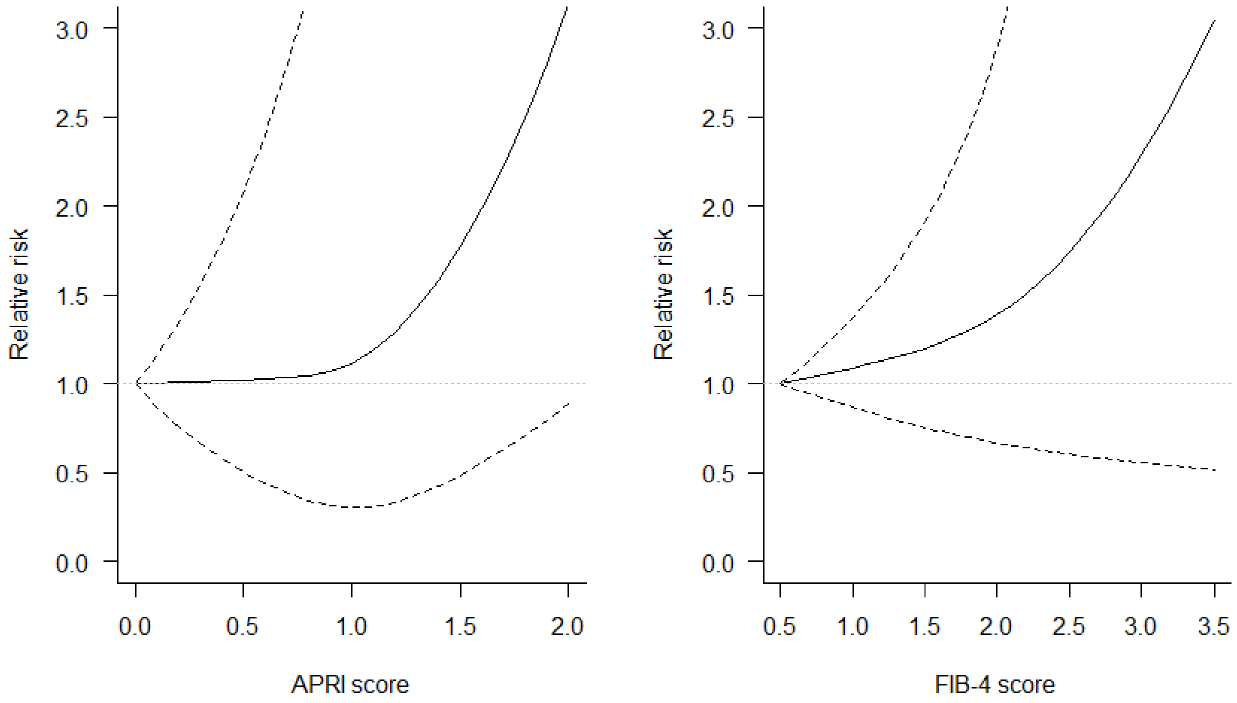

Supplement: Supplementary file 1 [file jcm-07-00219-s001.pdf]
